# Development and assessment of a questionnaire for a descriptive cross – sectional study concerning parents' knowledge, attitudes and practises in antibiotic use in Greece

**DOI:** 10.1186/1471-2334-9-52

**Published:** 2009-05-04

**Authors:** Sotiria G Panagakou, Maria N Theodoridou, Vassiliki Papaevangelou, Panagiotis Papastergiou, George A Syrogiannopoulos, Georgia P Goutziana, Christos S Hadjichristodoulou

**Affiliations:** 1 Department of Hygiene and Epidemiology, Faculty of Medicine, University of Thessaly, Thessaly, Greece; 2 First Department of Paediatrics, Agia Sofia Children's Hospital, University of Athens, Athens, Greece; 3 Athens School of Public Health, Department epidemiology Medical Statistics, Athens, Greece; 4 Department of Pediatrics, University of Thessaly, Faculty of Medicine, General University Hospital, Larisa, Greece

## Abstract

**Background:**

Upper Respiratory Infections (URIs) are common in children. The cause is usually viral, but parents' attitude often contributes to inappropriate antibiotic prescribing, promoting antibiotic resistance. The objective is to describe the process of developing a questionnaire to assess parents' Knowledge, Attitude and Practices (KAP) concerning the role of antibiotics when children suffer from URIs, as well as to evaluate the response rates, the completeness and the reliability (Cronbach) of the questionnaires. Finally, to note any limitations of the study.

**Methods:**

Literature review, along with pre – testing yielded a questionnaire designed to assess the parents' KAP – level. A postal survey was set, in a national sample of 200 schools stratified by geographical region. The participants consist of a multistage geographical cluster sample of 8000 parents. The influence of demographic characteristics (i.e. sex, age, education) was analyzed. Cronbach index test and factor analysis were used to assess the reliability of the questionnaire.

**Results:**

The response rate of the parents was 69%. Islands presented the lowest response rate while in Northern Greece the response rate was the highest. Sixty – eight point nine percent of the sample returned questionnaires fully completed, while 91.5% completed 95% of the questions. Three questions out of 70 were answered in a very low rate which was associated mostly with immigrant respondents. The section describing parents' attitude toward antibiotic use was not completed as much as the sections of knowledge or practices. The questions were factor analyzed and 10 out of the 21 extracted factors were finally evaluated, reducing the number of independent variables to 46. The reliability of the questionnaire was 0.55. However, only items that increased the Cronbach when added were eventually included in the final scales raising the internal consistency to 0.68. Limitations of the study, such as the vocabulary and form of the questionnaire and the idiocycrancy of the respondents, emerged during the analysis.

**Conclusion:**

The response rate and the completeness of the questionnaires were higher than expected, probably attributed to the involvement of the teachers. The study findings were satisfactory regarding the development of a reliable instrument capable to measure parents' KAP characteristics.

## Background

There has been strong evidence that antibiotic overuse or misuse has been correlated with increasing bacteria resistance [1-7]. In primary paediatric practice, a very frequent reason of antibiotic prescription is due to Upper Respiratory Infections (URIs) as children suffer from more than five URIs per year [8]. In England for example, approximately 70% of all antibiotics are prescribed for URIs [9,10]. Antibiotic resistance places a serious burden on the health economy since infections due to resistant bacteria require more specific and expensive antibiotics in order to heal. Additionally, such infections are are often related to extended hospital stays resulting in school absence for the students and possible leave of work for the parents [11].

According to the most recent results of ESAC (European Surveillance of Antimicrobial Consumption), during the period 2002 – 2004, Greece has been reported as the country with the most extended use of antibiotics [12]. Meanwhile, studies have shown worryingly high percentages of bacteria resistance in Greece. For example, in 2003, about 50% of the examined Staphylococci were found to be MRSA (Methicillin Resistant Staphylococci Aureus) placing Greece first in Europe [13]. In another study in 2002, 9% of the examined staphylococci turned to be resistant to vancomycine placing Greece fifth in the world [11]. Such results trigger the investigation for possible explanations involning this phenomenon. It has been cited in other articles that parents' attitudes and expectations may pressure pediatricians to prescribe more antibiotics more than necessary [14-18]. However, no such evidence has yet been proved in Greece.

In an attempt to understand the pediatrician's antibiotic prescribing patterns as well as the parents' demands in Greece, two KAP (Knowledge – Attitude – Practice) studies have been designed [19]. The KAP study by definition examines the Knowledge, Attitude towards and Practices concerning antibiotic use in these two groups [20]. In addition, the study was controlled by demographic data. Generally, most KAP studies are cross-sectional and employ a randomly selected population. The questionnaire is usually designed to create a relatively straightforward process for data collection, entry and analysis [21]. The present paper only describes the methodological issues of the parents' KAP study and questionnaire.

A comparative analysis of both KAP – studies (parents – pediatricians), however, should be conducted in the future. The results could be used to design a strategy on antibiotic resistance in Greece including intervention studies and campaigns. Besides, there is supportive evidence from other countries, such as Finland, which demonstrates that following strategic intervention programs, the use of several antimicrobial agents were significantly reduced [22].

Methodological issues have been emerged through the process of the study, and efforts have also been made to evaluate the extent in which the parents' KAP actually measures the knowledge, attitude and practice of the sample at a local and national level. In this paper the methodology pursued is being described, as well as the identification of the reliability and internal consistency of the questionnaire designed for the survey.

## Methods

### Questionnaire and KAP – study design

The main objective was to include questions on international experience, based on the methodology of KAP studies and adapted to the Greek culture and customs [19].

The KAP – questionnaire except for the demographic data is structured in three main sections: Knowledge concerning antibiotics (section A), attitude concerning the use of antibiotics (section B), and practise concerning the use of antibiotics (section C). A more detailed description of the content of each section is shown in Table 1.

**Table 1:** Detailed description of the final questionnaire form

Parts	Description
Demographic data	Age, sex, socio – economical status, education level, family status, insurance, number of children, region of living, possible immigration, e.t.c.
Section A (knowledge)	Differing antibiotical from symptomatical therapy, defining when parents would ask for antibiotics in case of a URI, stating if antibiotics have side – effects, or if they think that it is easy for the pharmaceutical society to produce consistently new antibiotic drugs.
Section B (attitude)	Questions like which symptom and after how long, would lead the parents to ask for antibiotic therapy, or how often and why they would use antibiotics without having consulted a paediatrician yet, are included. Other questions, such as if they would seek for a paediatrician who is more lenient with antibiotic administration, or whether they think that parents' are poorly educated by health campaigns in antibiotic use.
Section C (practises)	Quantity of antibiotic parents think that their child consumes compared with other children, or how often they praise the pediatrician who does not administer antibiotics. Furthermore, how often they obtain antibiotic after the pediatrician has approved it by phone without having previously examined the child, or how often they insist on taking antibiotics from the pediatrician, and finally how often the latter explains thoroughly the condition of the child and its pharmaceutical therapy.

To accomplish this goal, manuscripts and published papers describing similar research and methodological issues were studied [23,24].

The study design and questionnaire was approved by the educational institute of the Ministry of Education and General Assembly of the Medical Faculty at the University of Thessaly (reference number: 401/15 – 02 – 06). The scientific team included one epidemiologist, two paediatric specialists on infectious diseases, one statistician and one MD researcher. KAP questionnaires from other countries were referenced in order to formulate the Greek questionnaire. Initially, the draft of the questionnaire included 77 questions but after the pre-testing was taken into account, only 58 questions remained in the final questionnaire. The final form can be reached through additional file 1.

In order to assure the clearness, accuracy and consistency of the questions, the questionnaire was pre-tested among 30 parents. The scientific team evaluated the consistency and understanding of the completed questionnaires from the pre-test group and after excluding and modifying some questions, the final draft was created.

The questions (apart from the demographic data) were in a format of 5 possible answers for each question (accepting only one right answer), according to the 5 – Likert scale. This answering model facilitates with the filling of the questionnaire, data entry and statistical analysis.

Table 2 (similar and contradictive questions). illustrates the consistency of the parents' responses. To achieve these results, certain important questions were repeated by dividing them into two categories. The first category contained three pairs of questions. Each pair was stated in a different way but implied the same idea. Moreover, in the second category there were three pairs of questions which contradicted each other. Therefore, each pair of questions required the opposite answer. The scientific team followed this repetitive process so that the respondents could not understand that they were being asked the same question.

**Table 2:** Similar and contradictive questions included in the questionnaire

Number of couple of questions	Similar questions	Contradictive questions
1	A) The most URIs because of their being of viral cause cannot be healed with antibiotics.	A) Would you consider changing your paediatrician because he/she prescribes antibiotics very often?
	B) Do you think that if a URI follows its natural course without being treated with antibiotic therapy is right?	B) Would you consider changing your paediatrician because he/she does not prescribes antibiotics often?
2	A) When a child has fever, antibiotics should be administered.	A) The most URIs because of their being of viral cause cannot be healed with antibiotics.
	B) Would you consider giving antibiotic to your child when it has fever?	B) A child who suffers from a URI, can more quickly be healed if it is treated with antibiotics on time.
3	A)How often does your paediatrician prescribe antibiotics only because you have asked for it?	A) Would you reuse an antibiotic that had been used in a previous URI, to treat a new outcoming URI by yourself?
	B)How often do you ask firmly your paediatrician to prescribe antibiotics for your child?	B) Do you usually follow the instructions of your paediatrician?

Each questionnaire was given to the parents accompanied by a letter expressing the importance of the subject and their co-operation in the study.

### Sampling

A school based stratified geographic clustering sampling was used to select a representative sample of students. The kindergarten and elementary schools of Greece were collected from the Ministry Of Education which had revised the vocabulary and form of the parents' questionnaires and approved the administration of the survey via the school system with the school directors. Based on the mean number of students in each class, the number of the needed geographical clusters was calculated to be 200. Stratification was conducted using the population of the five regions of Greece (Peloponnesus, Islands, Athens, Northern Greece, and Central Greece). In total, 100 kindergarten and 100 elementary schools were randomly selected and stratified according to the population of each region.

The kindergarten (age 5) and first year students (age 6) were asked to participate in the study by their informed teachers. Their participation required them to provide their parents with the questionnaire for completion.

The teachers of each class were contacted by the researchers and received the appropriate guidance for the distribution of the questionnaires. Thus, the teachers better understood their responsibilities and the importance of the research. Each teacher made sure to provide the students with the questionnaire, collect the completed forms and return them to the researchers by mail. Finally, the teachers received an acknowledgement letter from the scientific team, thanking them for their contribution to the study.

### Statistical analysis

The data were entered in a database created by the Epi Info software. The statistical analysis [25,26] was conducted using the Epi Info [27] and SPSS software version 11.0. For quantitative data, the t – test was used, while for qualitative data the chi – square test was used. Statistically significant differences were considered when P value < 0.05. In descriptive analysis, univariate analysis was first used and afterwards the variables, which were statistically significant, were included in a logistic regression model. A five percent level of significance was adapted for testing analysis. The results were initially analysed with the principal component analysis method and were rotated with the variance maximising (varimax) system [25]. Cronbach's alpha is the numerical coefficient of reliability used in the study.

Factor analysis was used and reliability was assessed in order to detect underline structure, to reduce variables and to measure the internal consistency of the questionnaires. The initial collection of candidate variables to enter in a particular factor was considered as a variable's having a factor loading > 0.50. The second criterion in order a variable to be included in a factor, was the maximizing of the Chronbach's alpha (> 50%). This ensured that the content of the questions included in the particular factor would be maximised. Finally, the content of the variables which belonged into the same factor had to underline the same construct, so that it would make sense their all together consisting a factor under a new name.

## Results

The overall aim was to send 8000 questionnaires to 200 schools. However, eight school directors refused the participation of their school to the study. Due to this, 192 schools (96% of schools) participated, accounting for 7704 completed questionnaires. Five thousand three hundred and twelve questionnaires were collected out of 7704 that were finally sent, representing a 69% response rate. The overall response rate, however, was 66.4% (5312 questionnaires out of the intended 8000). Furthermore, the response rates differed among the Greek regions. As shown in Table 3, the highest response rate was in Northern Greece while the lowest was described on the Islands.

**Table 3:** Response rates within the Greek regions

AREAS	ATHENS	NORTHERN GREECE	CENTRAL GREECE	ISLANDS	PELLOPONESUS	TOTAL
Number of questionnaires sent	1995	2562	1140	1031	972	7704
Number of questionnaires collected back	1280	1905	783	642	702	5312
Response rate	64.16%	74.36%	68.68%	62.27%	72.2%	68.95%

Table 4 shows the relation between the response rate and the demographic characteristics in each region. As a result, parents who lived on the Islands portrayed the following characteristics: young age, low educational status, relatively low income, village or small town residents, parents of more than 3 children and immigrants status. These characteristics may contribute to the reduction of the response rate.

**Table 4:** Risk factors related to response rate within the Greek regions

AREAS	ATHENS	NORTH GREECE	CENTRAL GREECE	ISLANDS	PELLOPONESE	P value
	
Responders' characteristics	YES (%)	YES (%)	YES (%)	YES (%)	YES (%)	
Mother	1007/1256 (80.2)	1506/1853 (81.3)	605/757 (79.9)	501/620 (80.8)	549/683 (80.4)	0.918
Age < 45 years	1054/1157 (91.1)	1577/1675 (94.1)	628/664 (94.6)	506/523 (96.7)	586/619 (94.7)	0.000*
Having insurance	1250/1257 (99.4)	1868/1873 (99.7)	767/769 (99.7)	630/631 (99.8)	689/690 (99.9)	0.438
Special insurances	333/760 (43.8)	884/1850 (47.8)	347/746 (46.5)	312/614 (50.8)	341/684 (49.9)	0.069
high educational status of father	969/1191 (81.4)	1399/1752 (79.9)	513/710 (72.3)	392/576 (68.1)	473/651 (72.7)	0.000*
high educational status of mother	1083/1251 (86.6)	1569/1870 (83.9)	599/762 (78.6)	467/627 (74.5)	552/683 (80.8)	0.000*
High or moderate income	1106/1215 (91)	1584/1820 (87)	644/744 (86.6)	530/606 (87.5)	581/669 (86.8)	0.006*
Immigrants	142/1234 (11.5)	144/1835 (7.8)	72/750 (9.6)	85/622 (13.7)	70/677 (10.3)	0.000*
Habitants of regional towns	1049/1229 (85.4)	1167/1872 (62.3)	347/772 (44.9)	339/637 (53.2)	307/687 (44.7)	0.000*
Having <3 children	1224/1268 (96.5)	1779/1895 (93.9)	718/770 (93.2)	590/631 (93.5)	659/696 (94.7)	0.005*
Single parents	87/1273 (6.8)	96/1889 (5.1)	38/768 (4.9)	35/634 (5.5)	45/695 (6.5)	0.2
Child with recurrent URIs	183/1239 (14.8)	300/1864 (16.1)	98/748 (13.1)	100/625 (16)	111/684 (16.2)	0.327
Unintimate paediatrician	891/915 (97.4)	1456/1490 (97.7)	604/619 (97.6)	514/529 (97.2)	579/589 (98.3)	0.743
Poor access to healthcare system	460/1245 (36.9)	794/1854 (42.8)	353/751 (47)	295/614 (48)	307/682 (45)	0.000*

Seventy questions (100% completeness) were answered by 3660 parents (68.9%), while 4860 respondents (91.5%) presented a high completeness of 95% of the questions. Finally, there were a few parents (n = 48, 0.9% of the sample) who did not respond to > 50% of the questions. These questionnaires could further be excluded from the data analysis, because of poor completeness.

After analyzing the completeness of the questionnaire, 3 questions were identified with a proportionately low response rate in comparison to the rest of the questions. These were the following:

1) "Antibiotics decrease the complications of a URI."

2) "What kind of therapy/ies would you expect from your paediatrician to suggest for your child when it suffers from a URI?"

3) "How often would you like your paediatrician to prescribe antibiotics for your child when it suffers from: cold, nose drainage, sore throat, cough, vomit, ear pain?"

Table 5 shows the results of the univariate analysis regarding the completeness of each of the above three questions in relation to the parents' demographic characteristics. It indicates that characteristics such as being a father or older than 45 years, not having satisfactory health insurance, low educational status, low or moderate income, village or small town residence, immigrant status, and poor access to health services are all related to poor completeness for the above three questions. However, after assessment of the regression analysis, only the variable of immigrant status remained into the model and expressed the completeness of the two first questions, while low educational status, older age and immigrant status were the variables remaining in the model for the last one.

**Table 5:** Risk factors for completeness (Risk Ratio) of the three questions and regression analysis (Odds Ratio)

Responders' characteristics	Question 1				Question 2				Question 3			
	
	YES (%)	NO (%)	Risk Ratio	Odds Ratio	YES (%)	NO (%)	Risk Ratio	Odds Ratio	YES (%)	NO (%)	Risk Ratio	Odds Ratio
Mother	3856/4000 (96.4)	926/965 (96)	1 (0.99–1.02)		3698/3926 (94.2)	845/920 (91.9)	1.02 (1–1.05)*		3060/3571 (85.7)	652/808 (80.7)	1.06 (1.03–1.1)*	
Age < 45 years	4045/4196 (96.4)	250/268 (93.4)	1.03 (1–1.07)*		3813/4087 (93.9)	241/263 (91.6)	1.03(0.99–1.06)		3241/3755 (86.3)	180/227 (79.1)	1.09 (1.03–1.16)*	1.27 (1.1–1.49)*
Having insurance	4778/4987 (95.8)	14/15 (93.8)	1.02 (0.9–1.16)		4571/4878 (93.7)	14/15 (93.8)	1 (0.89–1.13)		3700/4389 (84.3)	12/14 (87.5)	0.96 (0.8–1.16)	
Special insurances	2077/2146 (96.8)	2173/2310 (94.8)	1.02 (1.01–1.03)*		1993/2102 (94.8)	2108/2267 (93)	1.02 (1–1.03)*		1664/1921 (86.6)	1658/2010 (82.5)	1.05 (1.03–1.08)*	
Father's high educational status	3481/3611 (96.4)	1023/1077 (95)	1.02 (1–1.03)*		3325/3530 (94.2)	970/1049 (92.5)	1.02 (1–1.04)*		2821/3250 (86.8)	711/898 (79.2)	1.1 (1.06–1.13)*	0.87 (0.78–0.98)*
mother's high educational status	3978/4122 (96.5)	800/859 (93.1)	1.04 (1.02–1.06)*		4790/4023 (94.2)	770/843 (91.3)	1.03 (1–1.05)*		3218/3707 (86.8)	503/681 (73.8)	1.18 (1.13–1.22)*	0.85 (0.75–0.96)*
High or moderate income	4106/4273 (96.1)	541/573 (94.4)	1.02 (1–1.04)*		3945/4188 (94.2)	506/555 (91.1)	1.03 (1–1.06)*		3258/3806 (85.6)	390/487 (80)	1.07 (1.03–1.12)*	
Immigrants	402/454 (88.5)	4304/4451 (96.7)	1.09 (1.06–1.13)*	0.43 (0.24–0.77)*	404/455 (88.7)	4078/4334 (94.1)	1.06 (1.03–1.1)*	0.6 (0.41–0.89)*	295/389 (75,8)	3343/3924 (85.2)	1.12 (1.07–1.18)*	2 (1.34–2.9)*
Habitants of regional towns	2945/3074 (95.8)	1816/1900 (95.6)	1 (1–1.01)*		2789/2992 (93.2)	1758/1869 (94)	0.99 (0.96–1.02)		1993/2336 (85.3)	1346/1636 (82.3)	1.04 (1.01–1.18)*	
Having <3 children	4569/4764 (95.9)	257/273 (94.1)	1.02 (0.99–1.05)		4353/4651 (93.6)	259/274 (94.5)	0.99(0.96–1.02)		3573/4213 (84.8)	170/222 (76.6)	1.1 (1.04–1.18)*	
single parents	270/285 (94.7)	4558/4753 (95.9)	0.99 (0.97–1)		263/281 (93.4)	4345/4642 (93.6)	1 (0.97–1.03)		213/253 (84.1)	3517/4177 (84.2)	1(0.95–1.05)	
Child with non recurrent URIs	4002/4182 (95.7)	747/769 (97.1)	0.99 (0.97–1)		3804/4077 (93.3)	722/756 (95.5)	0.98 (0.96–0.99)		3117/3689 (84.5)	572/673 (85)	0.99 (0.96–1.03)	
relative paediatrician	88/93 (94.9)	3729/3884 (96)	0.99 (0.94–1.03)		85/91 (93.7)	3522/3791 (92.9)	1.01 (0.96–1.07)		77/87 (88.8)	2860/3401 (84.1)	1.06 (0.98–1.13)	
Poor access to healthcare system	2746/2840 (96.7)	1993/2098 (95)	1.02 (1–1.03)*		2600/2763 (94.1)	1934/2067 (93.6)	1.01 (0.99–1.02)		2136/2505 (85.3)	1547/1848 (83.7)	1.02 (1–1.04)*	

Each questionnaire has been divided into three sections (apart from the demographic characteristics). The completeness of each section was also studied. All the questions in Section A (knowledge) were answered by n = 4592 responders (86.4% of the sample), while in section B (attitude) the number was reduced to n = 3802 (71.6%). In section C (practise), the number of the responders who answered all the questions was n = 4908 (92.4%). An association was identified between the demographic characteristics (referring mostly to responders with not satisfactory health insurance, low educational status, low or moderate income, immigrants and poor access to healthcare system) and the completeness of each section, as shown in Table 6. After the regression analysis, poor completeness of section A and section C was only associated with the fact of being an immigrant, whereas in section B the remaining variables expressing the completeness were the older age, were older age, poor health insurance, low educational status and immigrant status.

**Table 6:** Risk factors related to completeness (Risk Ratio) of each questionnaire section and regression analysis (Odds Ratio)

Responders' characteristics	Section A				Section B				Section C			
	
	YES (%)	NO (%)	Risk Ratio	Odds Ratio	YES (%)	NO (%)	Risk Ratio	Odds Ratio	YES (%)	NO (%)	Risk Ratio	Odds Ratio
Mother	3632 (87.6)	839 (83.8)	1.05 (1.02–1.08)*		3067 (73.6)	676 (67.5)	1.09 (1.04–1.14)*		3876 (93)	931 (93.1)	1 (0.98–1.02)	
Age < 45 years	3830 (88)	243 (84.7)	1.04 (0.99–1.09)		3232 (74.3)	183 (63.8)	1.17 (1.07–1.27)*	1.14 (1.01–1.28)*	4072 (93.6)	266 (92.7)	1.01 (0.98–1.04)	
Having insurance	4516 (86.8)	12 (75)	1.16 (0.87–1.54)		3748 (72)	10 (62.5)	1.15 (0.79–1.68)		4824 (92.7)	13 (81.3)	1.14 (0.9–1.44)	
Special insurances	1949 (87.9)	2081 (85.4)	1.03 (1.01–1.05)*		1658 (74.8)	1704 (69.9)	1.07 (1.03–1.1) *	1.06 (1.01–1.11)*	2099 (94.7)	2218 (91)	1.04 (1.02–1.06)*	
Father's high educational status	3282 (87.6)	968 (85.4)	1.03 (1–1.05)*		2826 (75.4)	736 (64.9)	1.16 (1.11–1.22)*	0.9 (0.82–0.98)*	3513 (93.8)	1022 (90.1)	1.04 (1.02–1.06)*	
mother's high educational status	3750 (87.8)	761 (82.4)	1.07 (1.03–1.1) *		3204 (75)	539 (58.4)	1.28 (1.21–1.36)*	0.83 (0.76–0.91)*	4007 (93.8)	814 (88.2)	1.06 (1.04–1.09)*	
High or moderate income	3892 (87.6)	510 (83.7)	1.05 (1.01–1.08)*		3281 (73.8)	386 (63.4)	1.16 (1.09–1.24)*		4153 (93.4)	548 (90)	1.04 (1.01–1.07)*	
Immigrants	380 (74.1)	4064 (83.3)	1.19 (1.13–1.26)*	2.3 (1.71–3.14)*	296 (57.7)	3384 (73.5)	1.27 (1.18–1.37)*	1.65 (1.22–2.2)*	420 (81.9)	4321 (93.8)	1.15 (1.1–1.19)*	3.04 (2.2–4.3)*
Habitants of regional towns	2788 (86.9)	1709 (86)	1.01 (0.99–1.03)		2335 (72.8)	1394 (70.1)	1.04 (1–1.08)*		2978 (92.8)	1826 (91.9)	1.01 (0.99–1.03)	
Having <3 children	4304 (86.6)	253 (87.2)	0.99 (0.95–1.04)		3590 (72.2)	190 (65.5)	1.1 (1.01–1.2) *		4600 (92.6)	271 (93.4)	0.99 (0.96–1.02)	
single parents	246 (81.7)	4315 (87)	0.94 (0.89–0.99)*		203 (67.4)	3574 (72.1)	0.93 (0.86–1.01)		272 (90.4)	4596 (92.7)	0.97 (0.94–1.01)	
Child with non recurrent URIs	3777 (86.5)	704 (88.9)	0.97 (0.95–1)		3141 (71.9)	586 (74)	0.97 (0.93–1.01)		4052 (92.8)	734 (92.7)	1 (0.98–1.02)	
relative paediatrician	83 (84.7)	3522 (87.1)	0.97 (0.89–1.06)		73 (74.5)	2907 (71.9)	1.04 (0.92–1.17)		93 (94.9)	3741 (92.5)	1.03 (0.98–1.07)	
Poor access to healthcare system	2610 (88.9)	1869 (84.6)	1.05 (1.03–1.07)*	0.84 (0.75–0.94)*	2143 (73)	1574 (71.3)	1.02 (0.99–1.06)		2751 (93.7)	2027 (91.8)	1.02 (1–1.04)*	

There were noted defaults in two multiple – answer questions. The first question was regarding the source from which parents obtained information on antibiotic use. Among the multiple answers there was also the option: "other". Here the responders could add a source they knew which was not already included in the multiple answers in first place. After the elaboration of the results, it was observed that 426 responders (8.02%) had added an extra source of information, while 126 of them to their job (health service) as the primary source. The same option was offered in the question asking parents "which possible treatment they expected for a URI, from their paediatrician." The results showed 225 parents (4.24%) who added another option while 181 of them mentioned "inhalers".

Table 7 shows the analysis results on how the questionnaires were completed (i.e. in a hurry or at random) by using similar or contradictive questions. According to the results, 11.9% of the questionnaires contained at least one answer that was not properly answered. The questionnaires (n = 48, 0.9%) which were poorly answered (<50% of questions) and the questionnaires that included at least 2 such responses (1.6% of the sample, n = 85) were further excluded from the data analysis. Thus, the number of questionnaires that were to be analysed was reduced from 5312 to 5179 (2.5% were excluded).

**Table 7:** Number of improperly answered questions

Number of improperly answered questions	Number of questionnaires	% of the sample
0	4680	88.1
1	547	10.3
2	71	1.3
3	13	0.2
4	1	0.0

### Factor analysis

Exploratory factor analysis of the questionnaire showed that items could be eliminated and still have a good amount of shared variance and sufficient coverage of each concept. The total number of variables studied was 70 and the factors that were related with variable groups were 21 (factors having eigenvalue >1.0). Only 10 factors were actually evaluated reducing the total of the independent variables to 46. Table 8 shows the final factors configured (renamed as separate variables) and their questions included as well as their factor loadings and the reliability associated.

**Table 8:** Results of factor analysis

Factors	Questions included	Factor loadings	Factor's reliability
1) How often would you like your paediatrician to prescribe antibiotics when your child suffers from a URI?	A) How often would you like your paediatrician to prescribe antibiotics when your child suffers from a common cold?	0.51	76%
	B) How often would you like your paediatrician to prescribe antibiotics when your child suffers from nose drainage?	0,52	
	C) How often would you like your paediatrician to prescribe antibiotics when your child suffers from a sore throat?	0,59	
	D) How often would you like your paediatrician to prescribe antibiotics when your child suffers from cough?	0,63	
	E) How often would you like your paediatrician to prescribe antibiotics when your child suffers from vomiting?	0,64	
	F) How often would you like your paediatrician to prescribe antibiotics when your child suffers from fever?	0,66	
	G) How often would you like your paediatrician to prescribe antibiotics when your child suffers from ear pain?	0,67	
2) Most URIs do not require antibiotic therapy to be	A) Most URIs, because of their being of viral cause, cannot be treated with antibiotics.	-0.72	74%
healed.	B) A child who suffers from a URI is healed more quickly if it is treated with antibiotics,	0.63	
	C) Do you think that if a URI follows its natural course without antibiotic administration is right?	-0.66	
3) How often would you give your child antibiotic without your paediatrician's prescription?	A) How often would you give your child antibiotic without your paediatrician's prescription because you did not have the money to pay the visit?	0.68	73%
	B) How often would you give your child antibiotic without your paediatrician's prescription because you did not think that it was serious enough to visit the paediatrician?	0.68	
	C) How often would you give your child antibiotic without your paediatrician's prescription because in the past your child had been treated with antibiotics for the same symptoms?	0.69	
	D) How often would you give your child antibiotic without your paediatrician's prescription because a pharmacist recommended it?	0.66	
	E) How often would you give your child antibiotic without your paediatrician's prescription because a friend – relative recommended it?	0.64	
4) Which one of the following is antibiotic?	A) Is amoxicillin antibiotic?	0.54	56%
	B) Is amoxicillin and clavulanic acid antibiotic?	0.55	
	C) Is erythromycin antibiotic?	0.62	
	D) Is cefouroxim antibiotic?	0.62	
5) Which one of the following is analgetic and antipyretic.	A) Is aketaminophene analgetic and antipyretic?	0.85	91%
	B) Is mefenamic acid analgetic and antipyretic?	0.87	
6) Do you agree that children should avoid taking antibiotics when not necessary?	A) Would you change your paediatrician because he/she gives antibiotics to your child very often?	0.3	52%
	B) Do you ask your paediatrician if it is actually necessary for your child to receive antibiotics?	0.72	
	C) Do you praise the paediatrician who prefers not to administer antibiotics to your child?	0.59	
7) Have you been informed about the judicious antibiotic use via the media?	A) Have you been informed about the judicious antibiotic use via the TV?	0.75	60%
	B) Have you been informed about the judicious antibiotic use via the radio?	0.68	
	C) Have you been informed about the judicious antibiotic use via the press?	0.79	
8) Would you visit a paediatrician because your child presented a symptom of a URI?	A) Would you visit a paediatrician because your child is coughing?	0.35	52%
	B) Would you visit a paediatrician because your child has a running nose?	0.63	
	C) Would you visit a paediatrician because your child has a sore throat?	0.48	
	D) Would you visit a paediatrician because your child has a roop?	0.64	
9) Would you expect from your paediatrician to treat a URI with antipyretic – analgesic drugs?	A) Would you expect from your paediatrician to treat a URI with aketaminophene?	0.81	61%
	B) Would you expect from your paediatrician to treat a URI with mefenamic acid?	0.82	
10) Have you been informed about the judicious antibiotic use from familiar persons?	A) Have you been informed about the judicious antibiotic use from relatives?	0.78	53%
	B) Have you been informed about the judicious antibiotic use from friends?	0.78	

The internal consistency for the summed rating scales was estimated using the Cronbach alpha coefficient. These estimates were based on the same sample that was applied for the exploratory factor analysis. The reliability of the questionnaire was measured and found to be 0.55. However, only items that increased the Cronbach alpha coefficient when added were eventually included in the final scales [28]. In this way, the reliability was raised to 0.68.

## Discussion

The overall response rate of the survey was 66.4%. However, given that 8 schools refused to participate, the response rate of the questionnaires that were finally distributed raised to 69%. This was higher compared to KAP – studies held in other countries where the response rate was lower (43% and 46% in two studies in Massachusetts, 59.3% in UK) [29-31]. Besides, in other KAP studies where the response rate was higher than in this research, the survey was conducted either by telephone (91.4% in Spain) or face – to – face interviews (80% in Boston, 86% in Los Angeles) [32,18,33]. Even in each Greek region separately, the response rate was still over 60%. This is particularly satisfactory given that the way the answers were derived from the parents was actually via mail. Usually, when potential responders are asked to return a filled questionnaire, they neglect to meet the requirements of the study because they do not undergo pressure i.e by an interviewer. In this research, as mentioned in the methodology, as soon as the parents filled the questionnaires, the latter were given to the teachers (through the students) who afterwards mailed them to the researcher. This indicates that the school based sampling was very helpful in achieving a high response rate, compared to the pattern of sending questionnaires directly to parents all over Greece, without their being enforced by school authorities.

Moreover, this kind of data collection was preferred versus the pattern of interviewing the parents, taking into account main drawbacks. First, the interviewer might influence the parents' response during their conversation, and secondly interviewees may respond in accordance with what they believe to be the "correct" replies. Additionally, the probability of the responders' embarrassment towards the interviewer would affect the quality of their answers. Moreover, a large number of interviewers would have to be trained to be sent to interview the parents, which was impractical. Finally, the variability among the interviewers could not be excluded. Using the questionnaires on the other hand, each responder received the same set of questions phrased in exactly the same way, so the answers were derived in a more objective way. Questionnaires may, therefore, yield data more precise than information obtained through an interview [21,34].

The significance of the topic was also clearly stated in an accompanying letter. Perhaps, this allowed the parent's to recognise the importance of the research and increased the response rate. An effort was also made to design a user-friendly questionnaire. It was as short as possible, clearly printed, defined important terms (i.e. URI) and categorised properly to promote easy and accurate responses. The questions were objective, with no leading suggestions to the desired answers [21,34].

The response rates differed among the Greek regions. Parents living on the Islands presented the lowest rate, while respondents in Northern Greece answered more frequently. The highest rate of a low educational status was noted on the islands, indicating that this could be a factor for the low response rate on the islands. In Northern Greece on the other hand, because the immigrants who were asked to fill the questionnaire were less than the immigrants of all other regions, it is more likely that most of the responders understood better the Greek language and the medical terms, upgrading in this way the response rate of this region. On the contrary, the Islands present the highest percentage of immigrants. Finally it is important to note that the response rate of each region is directly related to the vital status and the idiosyncrasy of each habitant and howsoever that influences it.

A high completeness was also noted with 91.5% of the parents answering to more than 95% of the questions. Considering that the content of the questionnaire may have been slightly difficult to understand due to the medical terminology, this was especially positive. The quality of the questionnaire was also assessed through the "similar or contradictive questions.". The parents who responded improperly to the above questions were few. Thus, the majority of parents filled the questionnaire with interest and caution, elevating in this way the quality of the responses.

Three questions, however, presented a very low completeness. The content of these three questions required that parents have specific knowledge about health issues. Unfortunately, the pre-tested results did not identify these questions, most likely due to the small number of parents included in the pre-test. According to the results, the parents who did respond to these questions tend to be mothers. This could be attributed to the fact that during their child's developing years they were more inclined to research information or had somehow been exposed to the subject of these questions. Moreover, these respondents were mostly younger than 45 years, possibly because their age group was better informed about the use of antibiotics. These parents also presented an elevated educational status that has offered them adequate information about health. Additionally, they were more likely to be city residents with high income and good health insurance. It is natural that such credentials can be related with elevated mental status and quality responses to these particular questions. Also, these respondents were mostly native Greek which for immigrants might have been harder to understand. Finally, they usually had satisfactory access to the health care system, which establishes the concern about their child's health. Besides, the health care system itself might provide them with information about antibiotic use and misuse. Overall, however, the regression analysis showed that immigrant status was mostly correlated to the low completeness of these three questions.

Concerning the analysis of the completeness of each section, it resulted that not all sections presented the same completeness. Most respondents tend to answer more to the section that referred to the parents' practises towards judicious antibiotic use. On the contrary, in section B (parents' attitude), the answers were the fewest. The demographic profile of the parents who did answer to all the questions in each section is in general the same which makes it difficult to understand any reason respondents were more likely to complete a section less or more than another. According to the regression analysis though, the variable of being an immigrant expressed the completeness of the sections the most. It can be suggested that, it was difficult to answer the hypothetical questions of attitude (Section B) because these parents may have not yet faced the situations that are stated in these questions. Additionally, the language of some of these questions contained specific terms. As far as section B is concerned, the regression analysis showed additional demographic characteristics associated to the completeness of this special section (older age, low educational status and poor access to health services) affecting in a multiple way the low completeness of this section. On the other hand, section C (practises) was much easier to complete since it simply requested approaches that parents practise in their daily routine and paediatrician visits. Section A (parents' knowledge), presented intermediate completeness compared to the other two sections.

The school environment played an important role in the procedure of the survey. Even though it had been permitted by the Ministry of Education, eight school – directors out of 200 (96%) refused their schools participation in the research. As a result, 300 questionnaires out of 8000 were not sent. However, this is an insignificant amount compared to the final 7700 questionnaires which were still delivered. Thus, the results were not likely to be altered significantly. Furthermore, the questionnaires that were decided not to be included for further analysis because of poor completeness or low interconsistency were few, not reducing in this way significantly the final amount of questionnaires to be analysed.

The main applications of factor analytic techniques are to reduce the number of variables and to detect structure in the relationship between variables, that is to classify variables [25]. During the factor analysis of the study, the variables were not limited significantly. This is satisfactory because it seems that most of the questions were put to derive certain information from each parent that no other question contained. There are two ways that reliability is usually estimated: test/retest and internal consistency (Cronbach index). Internal consistency estimates reliability by grouping questions in a questionnaire that measure the same concept. A basic limitation of the research was that the process of the study did not contain the test – retest process in order to estimate the reliability. Even though an attempt was made to conduct test/retesting, the candidate parents refused to participate, stating that they had already undergone this procedure once. As a result, it is hard to ascertain if the same replies would have existed for the same questions if the questionnaires had been readministrated to the same parents.

The reliability (internal consistency) of the whole questionnaire was 0.55. Reliability generally estimates the level in which all the variables measure the same construct. Variables derived from test instruments are declared to be reliable only when they provide stable and reliable responses over a repeated administration of the test [28]. Cronbach's alpha is the numerical coefficient of reliability used in the study. Alpha coefficient ranges in value from 0 to 1 and may be used to describe the reliability of factors extracted from dichotomous or/and Likert – scale formated questionnaires [28,35,36]. Cronbach alpha index is widely used in several statistical studies, including medical nature. The higher the score, the more reliable the generated set of questions. When data have a multidimensional structure (as in this study), Cronbach's alpha will usually be low. Zero point seven is supposed to be an acceptable reliability coefficient but lower thresholds are also used in bibliography [28,35,36]. Although the reliability of the questionnaire was measured, in this study, it is not representative becase the questionnaire was divided into three sections with different underline structures. Additionally, given that the number of response category answers following each question was rather small (5 – Likert scale), the resolution of the answering scales also remained small and therefore, the reliability was not significantly increased [36]. In this questionnaire though, it would be impractical to set longer answering scales because the respondents might get confused. However, the reliability was raised to 0.68 since only items that increased the Cronbach alpha coefficient, when added, were eventually included in the final scales [28].

Another limitation to the study was the language used to form the questions. A number of parents belonging to some minorities (mostly Gipsies, Albanians and Mouslims), either did not fill the questionnaires or gave inaccurate answers due to the language barrier or use of medical. Reversely, Greek parents might not understand Latin characters and could not reply to special questions that asked about common commercial names of certain substances. Additionally, it is important to mention that many parents might not understand similarly the clinical term of: upper respiratory infections. However, for the purpose of the questionnaire, a definition had been recorded, including various symptoms and diseases. Although there had been an effort to avoid complicated medical terms, this was not achieved 100% as regarded from the results. Thus, some parents might have misunderstood the term. Besides, there are diseases such as pharyngitidis or otitis, which might in fact need antibiotic therapy. The distinction between bacterial and viral infections might also complicate the responders.

It was noted during the elaboration of the results, that a lot of parents had added an extra answer in the option "other" in two multiple – answer questions (extra option "job" for question 14 and an extra option "inhalers" for question 24). These two answers were added in a significantly high rate and were not included in the multiple – answer questions a priori, as it should be. This was considered a limitation to the questionnaire design. Even though a pre – test was held initially, such possible answers were not revealed, explaining the reason of their absence in the final questionnaire. Again, indicating that a larger number of parents should have been originally pre – tested. In a future version of the questionnaire, for the question 14, the extra option "job" should also be included, while for the question 24, the extra option "inhalers" should be also included.

Another important concern was about differences between those who decided to respond or not. It might be suggested that non – responders respondents felt that they strongly believed their own knowledge concerning antimicrobial use, neglected to complete the questionnaire. Low socioeconomic and un-educated respondents or others who are not aware of the subject may have hesitated to answer in fear of embarrassment.

Another limitation to the study was that among physicians there were differences in prescribing habits. This means that in some occasions the parents' answers might be influenced by their pediatricians' KAP – level. So the parents' aspect cannot be evaluated as accurately. It is possible that many parents might have endorsed their physician as their primary health influence and derived most of their opinions regarding antibiotics from them. In this case, an advantage arises, as a general idea of the pediatricians' KAP – level is being emerged.

At this point, it should be mentioned that the parents' answers might have been affected by the time of year they filled the questionnaire. Given that statistically, the peak use of antibiotics is observed especially between January and March. According to these statistics, it can be assumed that it is possible that the parents who filled the questionnaire during this period, might have described incorrectly a practice closer to antibiotic overuse. Moreover, the answers may depend on how susceptible the child is to URIs. After all, the more often and the more serious the symptoms are, the more likely parents might seek treatment with antibiotic.

One question asks how many days the parents should wait before visiting a pediatrician in case their child suffers from any symptoms of URIs. Unfortunately the kind of symptom is not specified. This means that some parents may think of a severe ear – pain or just a mild nose drainage which would hardly demand a visit to the pediatrician, disfiguring the answer. Finally, the following questions which ask whether the parents think that they worry about their child more than other parents, how much they think they are informed about judicious antimicrobial use, and if they think that their child gets more antibiotics in relation to other kids, require highly subjective answers. Such answers might be difficult to be evaluated because the standards of each parent differ and thus, cannot be estimated or compared.

## Conclusion

In the present research the response rate was satisfactory and the content of the questionnaires was described with high quality. These two factors contribute in revealing as much as possible the level of knowledge, attitude and practice of the parents in Greece. The reliability of the questionnaire was also satisfactory, given the particularities of the form of the questionnaire.

Yet, all such studies, even those with considerable flexibility in their design, describe only a reported KAP profile, which is usually ideal or typical rather than actual. Bearing the limitations in mind, however, the study offers insight for the public health community and health officials about their role, while testing the efficacy of a modified KAP questionnaire on the public. The results may influence future implementation programs on antibiotic use, helping reduce their misuse.

## Competing interests

The authors declare that they have no competing interests.

## Authors' contributions

CSH, MNT, EP, GAS and SGP designed the study. SGP, PP and MK collected the data. CSH, SGP, PP and MK performed the statistical analysis and interpreted the results. CSH and SGP wrote the manuscript. CSH, GAS, MNT and EP provided valuable insight for revising the manuscript. All authors read and approved the final manuscript.

## Pre-publication history

The pre-publication history for this paper can be accessed here:

http://www.biomedcentral.com/1471-2334/9/52/prepub

## Supplementary Material

Additional file 1 **Questionnaire**. Final form of the questionnaire. Click here for file
