# Supplementary material for: Development and assessment of a questionnaire for a descriptive cross – sectional study concerning parents' knowledge, attitudes and practises in antibiotic use in Greece
Source: BMC Infect Dis. 2009 May 4;9:52. doi: 10.1186/1471-2334-9-52 (PMC2686701; doi:10.1186/1471-2334-9-52)
Supplement: Additional file 1 — Questionnaire. Final form of the questionnaire. [file 1471-2334-9-52-S1.doc]

***QUESTIONNAIRE***

**Demographic characteristics**

1. Sex: Male

Female

1. Age: ____ years old
2. Assurance: Public

Private

Both

None

1. Type of public insurance:____________

1. Parents’ educational status:

|  | Mother | Father |
| --- | --- | --- |
| Primary School |  |  |
| Secondary School |  |  |
| High School |  |  |
| College |  |  |
| University – Postgraduate studies |  |  |

1. Would you describe your

| Very high | High | Μoderate | Low | Very Low |
| --- | --- | --- | --- | --- |
|  |  |  |  |  |

family income level as:

1. Are you immigrant in Greece? No

Yes Native country: ____________

1. Are you habitants of: Big town

Small town

Village

1. Number of children: ____
2. Are you a single parent? No

Yes

1. Does your child suffer often from Upper Respiratory Infections (such as colds, ear infections, sore throats)? Yes

No

1. Does your pediatrician happen to be: Family relative

Friend

Just professional relation

1. Would you consider your

| Very good | Good | Μoderate | Bad | Very bad |
| --- | --- | --- | --- | --- |
|  |  |  |  |  |

access to health services as:

**Section A**

1. Sources of information you have about judicious antibiotic use:

Physician

Television

Radio

Newspaper

Friend

Family relative

Other: ___________

1. Which one of the following drugs are antibiotics?

Augmentin

Depon

Aerolin

Ceclor

Ponstan

Amoxill

Mucosolvan

Erythrocin

|  | Strongly agree | Agree | Uncertain | Disagree | Strongly disagree |
| --- | --- | --- | --- | --- | --- |
| 1. Antibiotic must be administered in any case, once a child has fever. |  |  |  |  |  |
| 1. As most of the Upper Respiratory Infections (like colds, flue, sore throats, ear infections) are of viral cause, they must not be cured with antibiotics. |  |  |  |  |  |
| 1. If a child suffers from a flue or a cold, it will be quirkier cured if it receives antibiotic on time. |  |  |  |  |  |
| 1. Scientists can always produce new antibiotics that are able to kill the resistant bacteria. |  |  |  |  |  |
| 1. Antibiotics do not present side - effects. |  |  |  |  |  |
| 1. When antibiotics are administered where there is no special reason, their efficacy is decreased and bacteria become more resistant. |  |  |  |  |  |
| 1. Antibiotics decrease the complications of an Upper Respiratory Infection. |  |  |  |  |  |

**Section B**

1. How many days would you let pass in order to visit a pediatrician, if your child presents some symptoms (ie. Nose drainage, sore throat, vomit, cough, fever)? ________days
2. What kind of therapy - ies would you expect from your pediatrician to suggest for your child when it suffers from an Upper Respiratory Infection?

Antibiotics

Analgesics – antipyretics

Antitussives

Antistamines

Normal Serum

Other: ________

1. Which one – s of the following symptoms would make you visit a pediatrician for your child?

Cough

Fever

Nose drainage

Ear pain

Sore throat

Hoarseness

Change of behavior

Other: __________

| 1. How often would you like your pediatrician to describe antibiotics for your child when it suffers from: | Always (95-100%) | Most of the times (70-95%) | Often (30-70%) | Some times (5-30%) | Never  (0-5%) |
| --- | --- | --- | --- | --- | --- |
| Cold |  |  |  |  |  |
| Nose drainage |  |  |  |  |  |
| Sore throat |  |  |  |  |  |
| Cough |  |  |  |  |  |
| Vomit |  |  |  |  |  |
| Fever |  |  |  |  |  |
| Ear pain |  |  |  |  |  |

| 1. How often would you give your child antibiotics without the pediatricians’ advice, for the following reasons? | Always (95-100%) | Most of the times  (70-95%) | Often (30-70%) | Some times  (5-30%) | Never (0-5%) |
| --- | --- | --- | --- | --- | --- |
| Ι) Because you did not have enough spare time to visit a pediatrician, or because you did not have enough money to pay the visit. |  |  |  |  |  |
| ΙI) Because you thought that your child’s condition was not serious enough. |  |  |  |  |  |
| III) Because your pediatrician had prescribed the same antibiotic in the past, for the same symptoms. |  |  |  |  |  |
| IV) Because a pharmacist recommended the antibiotic. |  |  |  |  |  |
| V) Because a friend/ family relative recommended the antibiotic. |  |  |  |  |  |

|  | Strongly agree | Agree | Uncertain | Disagree | Strongly disagree |
| --- | --- | --- | --- | --- | --- |
| 1. Do you believe antibiotics are used too much? |  |  |  |  |  |
| 1. Would you change your pediatrician because according to your opinion he/she does not prescribe antibiotics often enough for your child? |  |  |  |  |  |
| 1. Would you change your pediatrician because according to your opinion he/she prescribes antibiotics for your child very often? |  |  |  |  |  |
| 1. Would you reuse an antibiotic which you had used in the past if your child presents the same symptom - s? |  |  |  |  |  |
| 1. Do you think that parents and pediatricians should be informed about judicious antibiotic use? |  |  |  |  |  |
| 1. Do you think that most of the Upper Respiratory Infections will be self – cured even without the use of antibiotics? |  |  |  |  |  |
| 1. Would you press your pediatrician for antibiotic therapy if your child suffers from recurrent Upper Respiratory Infections? |  |  |  |  |  |
| 1. Would you visit a pediatrician just because of nose drainage? |  |  |  |  |  |
| 1. Do you think that you worry about your child more than other parents do for theirs? |  |  |  |  |  |
| 1. Would you visit a pediatrician in order to avoid any complications of your child’s infection? |  |  |  |  |  |

**Section C**

|  | Very much | Plenty | Not much | A little | None |
| --- | --- | --- | --- | --- | --- |
| 1. How much do you think that you are informed about judicious antibiotic use? |  |  |  |  |  |
| 1. How much antibiotic do you think your child receives compared to other children? |  |  |  |  |  |
| 1. How much do you pay attention to the possible side – effects of antibiotics? |  |  |  |  |  |
| 1. Do you agree that you will be dissatisfied if your pediatrician does not prescribe an antibiotic for your child’s Upper Respiratory Infection? |  |  |  |  |  |

|  | Always (95-100%) | Most of the times  (70-95%) | Often (30-70%) | Some times  (5-30%) | Never (0-5%) |
| --- | --- | --- | --- | --- | --- |
| 1. In case your pediatrician prescribes an antibiotic, how often do you ask him – her if it is actually necessary? |  |  |  |  |  |
| 1. How often do you praise your pediatrician if he/ she prefers not to prescribe antibiotic? |  |  |  |  |  |
| 1. How often does your pediatrician recommend antibiotic therapy by phone? |  |  |  |  |  |
| 1. In case you strongly wish your child to receive antibiotic, how often do you ask for it directly to the pediatrician? |  |  |  |  |  |
| 1. How often do you follow all the pediatrician’s instructions and advice? |  |  |  |  |  |
| 1. How often do you urge your pediatrician to prescribe antibiotic even when the diagnosis is not confirmed? |  |  |  |  |  |
| 1. How often does your pediatrician explain to you about your child’s condition and if it should or shouldn’t receive antibiotics? |  |  |  |  |  |
| 1. How often do you think that your pediatrician prescribes antibiotic only because you asked him to? |  |  |  |  |  |
